# Pieces of the puzzle: The opportunities and challenges of integrative learning systems for patient safety

**DOI:** 10.1177/13558196211050541

**Published:** 2021-12-08

**Authors:** Justin Waring

**Affiliations:** Health Services Management Centre, 151150 University of Birmingham, Birmingham, UK

It is a little over 20 years ago that ‘patient safety’ became a substantial policy priority.^1^ Clearly mistakes, mishaps and iatrogenic harms are as old as medicine itself, but throughout the 1990s, patient safety emerged as a distinct research and policy domain fuelled by a growing body of research, high-profile scandals of service failure and the publication of reports such as *To Err is Human*^2^ and *An Organisation with a Memory*^3^*.* One prominent idea developed through these reports was that despite there being a range of legal, professional and organisational frameworks for dealing with issues of clinical risk, there was little in the way of a dedicated or systematic approach to learning and improvement. Borrowing from other high-risk industries, notably aviation, health care systems across the world set about introducing incident reporting and investigation systems, such as the National Reporting and Learning System in the English National Health Service.

As Van Dael and colleagues^4^ rightly note, there is an abundance of research demonstrating the shortcomings of reporting systems, most notably associated with professional and organisational cultures, as well as other more technical or practical barriers.^5^ The premise of their paper is that safety issues might be better identified and analysed through combing multiple sources of information, which, in the case of their study, include staff incident reports with the formal complaints made by service users and their relatives. As they describe, each offers an important, albeit incomplete, part of the picture, and so, to what extent can these sources be brought together to inform improved learning? Their study notes some important differences between these information sources. Staff reports are shaped by local working cultures and norms, whereas patient complaints are guided by different expectations, norms and beliefs. In turn, staff reports tend to focus on individual events in terms of more narrow clinical issues, whereas complaints tend to describe the escalation of events across time and space in terms of broader contributory factors. Their study shows that there is moderate agreement between staff incident reports and patient complaints around mutually identified events; but, as expected, there are important differences. The extent of mutuality seems to relate to the degree of severity or significance, that is, both staff and patients are more likely to report the same event if it results in significant harm. Where common or mutual reports are identified, it is not necessarily the case they will complement each other. Complementary accounts more often describe common events; but contested accounts are characterised by different attributions of responsibility. This is hardly surprising. It might be expected that multiple parties will witness or identify a significant safety event, but these parties will hold differing views of why this event came about.^6^

What Van Dael et al.’s study demonstrates is the possibility for better understanding the occurrence, causes and impacts of safety events through integrating multiple sources of information, insight and intelligence. As well as incident reports and complaints, this could include the outcomes of clinical audits and mortality reviews, the statistical analysis of routinely collected service data, and more explicit and transparent use of ‘soft intelligence’.^7^ The point is that each source shines a light of the problem of safety, but each leaves areas in the dark, and therefore, through combining multiple sources, we better understand the issues. However, there are important qualifications to this idea, especially where we run the risk of producing a confusing mosaic that hinders improvements.

I use the alliterative phrase ‘information, insight and intelligence’ to acknowledge that each source offers a distinct way of ‘knowing’ or expressing ‘knowledge’ about patient safety.^8^ In some regards, the sources of knowing come in the form of ‘hard’ facts and data that are more readily codified and communicated,^9^ but in other regards, they come in the form of ‘soft’ insight and emotive feeling that can be difficult to articulate or codify.^7^ Whilst early policies focused on formal reporting procedures, there has since been attention to the opportunities and safeguards for those who wish to speak-up about safety issues.^10^ However, the challenge remains as to how to bring together and reconcile the different epistemologies of safety in ways that does not assume the superiority of one over the other.

A further point for consideration is that each source of information or insight is conceived with a particular purpose and is anchored within a given socio-cultural system of governance.^9^ Incident reporting and complaints systems are clearly designed to address different purposes, and each is situated within particular regulatory systems that imply different notions of accountability, responsibility and justice. Although there is scope to bring these together, it is important to recognise that they gather information to address distinct objectives and those who engage with these systems often do so for different reasons. This inevitably means there will be tensions. Equally, but less obviously, the softer insights of safety are framed by the distinct cultural norms and values of epistemic communities, where understandings of risk and responsibility often function as a basis for group identification and differentiation. That is, how we think about safety relates to how we think about ourselves and others. As such, questions of blame and accountability are often integral to these insights.^5^ The point is that diverse, and possibly competing, values and norms underpin the ways people make sense of and communicate their experiences of safety, and that these are not always easy to reconcile.^11^

Finally, and in more practical terms, Van Dael et al's paper highlights the possibility of combining and integrating incident reports and complaints, but it is relatively silent on how this can be done in a systematic or routine way outside of a research study. Taking into account the above issues, the work of translating and integrating different sources of information, insight and intelligence in any systematic way seems especially challenging. At best, some targeted integration of information systems, perhaps drawing on advances in computational science, is one way of integrating and analysing different ‘data’ sources, but then the challenge will be how to integrate with other forms of knowing – in other words, bridging the gap from ‘knowledge management’ to individual and collective understanding and action.^12^ Existing healthcare governance procedures provide an obvious platform for this, such as board meetings or clinical audit committees, but the challenge then becomes how to translate and integrate different sources of knowledge.^7^ Research shows, for example, that the translation and brokering of knowledge between epistemic communities can engender more comprehensive and targeted safety improvements.^8^ But this often relies upon sustained and situated interaction to appreciate the underlying meanings and values inherent to different epistemologies of safety. Of course, existing governance structures tend to involve communities that are professionally, culturally and epistemologically narrow. A long history of research in organisational psychology highlights the challenges posed by homogeneity – and the advantages proffered by more diverse boards and committees. A key practical challenge, therefore, is ensuring that the richness, the breadth and even the inconsistencies of the knowledge available to decision-makers are not ‘processed out’ by those making sense of them. How best to do this – for example, by diversifying the membership of committees at every level or by opening up meetings to outsiders, other occupational groups or patients – is a question ripe for innovation, research and evaluation.
